# Correction of fetal umbilical vein flow imbalance following laser surgery for twin–twin transfusion syndrome

**DOI:** 10.1002/uog.26041

**Published:** 2022-12-01

**Authors:** R. H. Saab, G. R. DeVore, M. Monson, J. Masri, L. M. Korst, R. H. Chmait

**Affiliations:** ^1^ Division of Maternal–Fetal Medicine, Department of Obstetrics and Gynecology Keck School of Medicine, University of Southern California Los Angeles CA USA; ^2^ Fetal Diagnostic Centers Pasadena CA USA; ^3^ Department of Obstetrics and Gynecology David Geffen School of Medicine, UCLA Los Angeles CA USA; ^4^ Department of Obstetrics and Gynecology Wayne State University Detroit MI USA; ^5^ Childbirth Research Associates North Hollywood CA USA

**Keywords:** fetoscopic laser surgery, TTTS, twin–twin transfusion syndrome, umbilical vein Doppler, umbilical vein flow

## Abstract

**Objectives:**

Twin–twin transfusion syndrome (TTTS) is characterized by unequal hemodynamics between the twins. We aimed to assess preoperatively the difference in umbilical vein flow (UVF) between the recipient and donor monochorionic diamniotic twins and evaluate the change in UVF following laser surgery in both twins.

**Methods:**

This was a retrospective cohort study of differences in UVF that occurred following laser surgical treatment of TTTS. Sonographic assessment of the umbilical vein before and 24 h after fetoscopic laser surgery for TTTS was performed. Umbilical vein diameter and time‐averaged maximum velocity were measured, and UVF per kg (UVF/kg) was converted into a *Z*‐score by a calculator created using gestational age as an independent variable. *Z*‐score values were converted into centiles, which were evaluated statistically. Median differences in UVF/kg centile values were adjusted for TTTS stage and presence of arterioarterial anastomoses.

**Results:**

The study population consisted of 363 TTTS patients. The adjusted preoperative median difference in UVF/kg centile between the recipient *vs* donor twin was 17.9% (−17.1% to 57.6%), *P* < 0.0001. The adjusted median difference in UVF/kg centile between the postoperative *vs* preoperative period among recipients was 2.2% (−10.8% to 13.8%), *P* < 0.0001, while the adjusted median difference among donors was 27.3% (8.2%–34.6%), *P* < 0.0001.

**Conclusion:**

The preoperative difference in UVF between the recipient and donor twins confirms the pathophysiology of TTTS. Postoperatively, the substantial increase in UVF of the donor twin and the relatively small increase in UVF of the recipient twin confirm that ablation of the vascular communications resulted in rapid improvement in perfusion of the donor twin. © 2022 The Authors. Ultrasound in Obstetrics & Gynecology published by John Wiley & Sons Ltd on behalf of International Society of Ultrasound in Obstetrics and Gynecology.

**CONTRIBUTION**

**What are the novel findings of this work?**

In this large study on monochorionic diamniotic twin pregnancies treated with laser surgery for twin–twin transfusion syndrome (TTTS), we employed a newly designed *Z*‐score calculator, which uses gestational age as an independent variable, to evaluate changes in umbilical vein flow (UVF) following treatment. We demonstrated a significant difference in the UVF between recipient and donor twins, confirming unequal intertwin blood flow as the cause of TTTS. We also observed a significant postoperative increase in UVF of the donor twin, confirming fetoscopic laser surgery as an effective treatment on a pathophysiological level.

**What are the clinical implications of this work?**

This study sheds light on the preoperative difference in UVF per kg between recipient and donor twins in TTTS. This paves the way for future studies to evaluate the role of this difference in UVF per kg in predicting TTTS, as a prediagnostic step or an estimate of severity.

## INTRODUCTION

Twin–twin transfusion syndrome (TTTS) occurs in approximately 10% of monochorionic diamniotic (MCDA) pregnancies, and carries a significant risk of perinatal morbidity and mortality if left untreated^1^. While the circulatory systems of MCDA twins are connected normally through vascular communications in the common placenta, it is the abnormal net shunting of blood from one fetus (donor) to the other (recipient) through these channels that results in TTTS^1^. Consequently, relative hypovolemia in the donor twin manifests as oligohydramnios and poor growth. The relative hypervolemia in the recipient is associated with polyhydramnios, fluid overload and heart failure. Quintero staging has been used to describe the severity of TTTS^2^.

Although different management options for TTTS have been described, fetoscopic laser ablation of communicating vessels remains the first‐line treatment for Quintero Stage‐II, ‐III and ‐IV TTTS^1^, ^3^, ^4^, ^5^, ^6^. There is no consensus regarding optimal management of Stage‐I TTTS^7^, ^8^, ^9^. Laser ablation is performed on all placental vascular communications, usually between 16 and 26 weeks of gestation^1^. This has been shown to result in a survival rate of 70–90% for at least one twin and 40–70% for both twins^3^, ^6^, ^10^, ^11^. Both the amniotic fluid volume in the donor and recipient sacs^12^ and the aberrant fetal blood flow^13^, ^14^, ^15^, ^16^, ^17^ are expected to normalize postoperatively.

Given that the umbilical vein (UV) is the only conduit of blood from the placenta to the fetus, assessment of its pre‐ and postoperative flow may help understand the pathophysiology of TTTS. Previous studies have demonstrated a significantly higher UV flow (UVF) of recipients compared with donors in TTTS^13^, ^14^, ^15^, ^16^, with correction of intertwin UVF discordance following laser surgery^13^, ^14^, ^15^, ^17^. To confirm these findings, the present study evaluated a large sample size of TTTS fetuses undergoing laser surgery using a newly designed *Z*‐score calculator that converts *Z*‐scores to centiles as a clinical tool to measure changes in UVF before and after laser surgery^18^.

The aims of this study were (1) to describe the difference in UVF between the recipient and donor in MCDA twin pregnancies complicated by TTTS prior to laser surgery and (2) to evaluate the change in UVF following laser surgery in both twins.

## METHODS

This was a retrospective cohort study of changes in UVF following laser surgical treatment of MCDA twin pregnancies complicated by TTTS. The study population consisted of all patients diagnosed with TTTS, including Stage‐I TTTS patients, who underwent fetoscopic selective laser photocoagulation of communicating vessels (laser surgery) between 18 and 26 gestational weeks at our center (Los Angeles Fetal Surgery, Keck School of Medicine, University of Southern California, Los Angeles, CA, USA) from February 2010 to May 2020. Laser surgery was offered routinely to patients with symptomatic or unstable Stage‐I TTTS (e.g. patients with symptomatic polyhydramnios or short cervix). Stable patients were counseled regarding the lack of consensus with respect to optimal first‐line management of stable Stage‐I TTTS, as detailed in a recent article^19^. Patients were then informed of perinatal survival rates of fetuses with Stage‐I TTTS at our institution^11^. The risks associated with laser surgery and alternative treatments were discussed, and written informed consent was obtained from patients who expressed desire to proceed with laser surgery after thorough counseling. Study exclusion criteria were higher‐order multiple pregnancy, monoamniotic twin pregnancy, gestational age (GA) < 18 and > 26 weeks, repeat or incomplete laser surgery, persistent or reversed TTTS following laser surgery, single fetal demise on postoperative day 1 and missing UV Doppler measurements in either twin at either preoperative or postoperative assessment. Patients with GA < 18 weeks were excluded for consistency with the reference population based on which the *Z*‐score calculator was computed (G. DeVore, pers comm). Incomplete laser surgery was defined as inability to perform laser photocoagulation of all intertwin vascular anastomoses. Reasons for incomplete surgery may include poor visualization of vascular anastomoses secondary to discolored amniotic fluid (e.g. due to pre‐existing subchorionic hematoma or bleeding from uterine trocar insertion), challenging placental location and maternal or fetal instability precluding ability to complete surgery. Each patient underwent a detailed preoperative ultrasound examination, which included evaluation of cervical length, amniotic fluid volume, fetal morphology, biometry, estimated fetal weight (EFW) and Doppler flow of the umbilical artery, UV and ductus venosus. The preoperative ultrasound examination was performed 1 day prior to surgery, and the postoperative scan was conducted on postoperative day 1. EFW was not remeasured postoperatively.

UV parameters were measured from the free loop of the UV at or near the abdominal wall insertion site. Color Doppler was activated to visualize flow and measure the time‐averaged maximum velocity (TAMX). The same section of the sampled UV, adequately zoomed, was used to measure the UV diameter (UVD) from the anterior inner to the posterior inner wall perpendicular to the beam of ultrasound (Figure 1). All angles of insonation were equal or close to 0°. Measurements with angles > 30° were rejected. Angle correction was performed as a last resort. No fetal breathing or movement was present at the time when measurements were taken.

**Figure 1:**
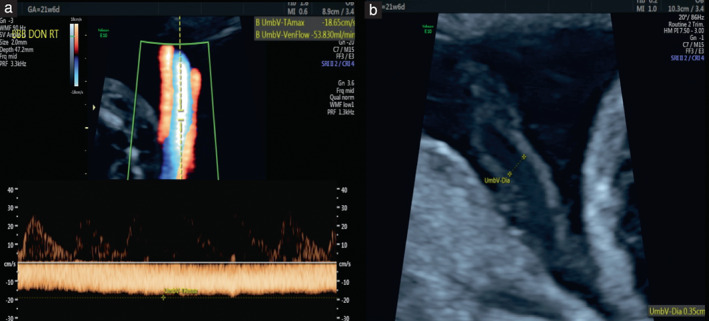
Sonographic method of measuring umbilical vein time‐averaged maximum velocity (TAMX) and diameter (UVD) at a free loop of the umbilical vein. (a) Pulsed‐Doppler waveform showing TAMX measurement of 18.65 cm/s. (b) Adequately zoomed longitudinal section of the umbilical vein with UVD measurement of 0.35 cm.

Assuming parabolic flow in a straight lumen, UVF (mL/min) was calculated based on the cross‐sectional area (cm^2^) and TAMX (cm/s) as follows: UVF = 3.14 × (UVD/2)^2^ × 0.5 × 60 × TAMX. UVF/kg was derived by dividing the UVF by the EFW in kg. UVF/kg *Z*‐scores and the corresponding centiles (UVF/kg centiles) were calculated using a *Z*‐score calculator with a range of 18–26 gestational weeks, created using GA as an independent variable in a population of normal singleton fetuses^18^.

Median differences between centiles were calculated. For Analysis 1, the difference of interest was between the recipient and donor in the preoperative period (recipient minus donor). This difference was also calculated for the postoperative period for completeness. For Analysis 2, the difference between preoperative and postoperative measurements was calculated separately for donors and recipients (postoperative minus preoperative). These differences were tested and found to vary with Quintero stage and presence of arterioarterial (AA) anastomoses on bivariate analyses (Tables 2 and 3); they did not vary according to presence of selective fetal growth restriction (sFGR) in the donor twin (data not shown). Consequently, the differences were adjusted for Quintero stage and presence of AA anastomoses in a linear regression equation to obtain predicted values of the centiles for these differences. The predicted values were then tested for a difference from zero using the Wilcoxon signed‐rank test.

Data are presented as *n* (%) or median (range). Analysis was performed using SAS statistical software (version 9.4; SAS Institute, Cary, NC, USA). The study was approved by the University of Southern California Health Sciences Campus institutional review board (HS‐09‐00690, HS‐05‐00374).

## RESULTS

Of 633 patients diagnosed with TTTS and treated with laser surgery during the study period, 270 were excluded, resulting in 363 patients included in the final study population (Figure 2). The median preoperative GA of this population was 20.7 weeks. The Quintero stage distribution for the population was as follows: 79 (21.8%) had Stage‐I, 93 (25.6%) had Stage‐II, 161 (44.4%) had Stage‐III and 30 (8.3%) had Stage‐IV TTTS. Table 1 summarizes the preoperative patient characteristics.

**Figure 2:**
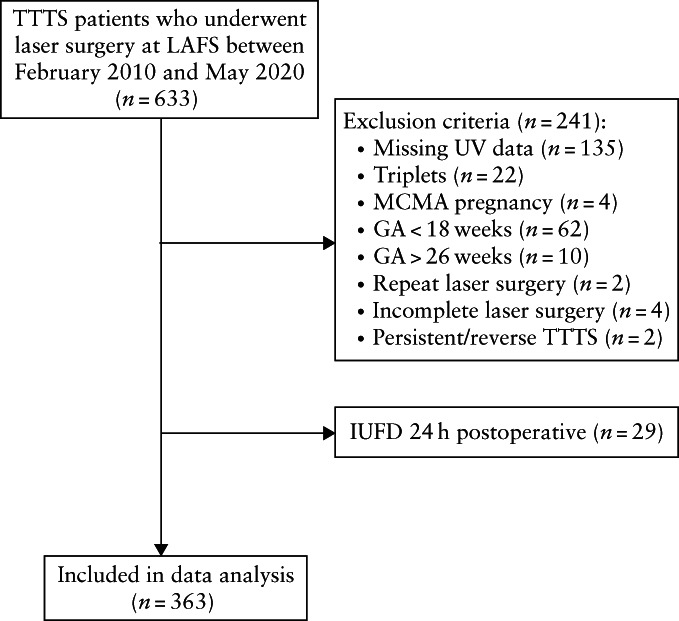
Flowchart summarizing inclusion in the study of monochorionic diamniotic twin pregnancies undergoing laser surgery for twin–twin transfusion syndrome (TTTS). GA, gestational age; IUFD, intrauterine fetal death; LAFS, Los Angeles Fetal Surgery; MCMA, monochorionic monoamniotic; UV, umbilical vein.

**Table 1:** Preoperative characteristics of 363 monochorionic diamniotic twin pregnancies complicated by twin–twin transfusion syndrome

Characteristic	Value
Preoperative gestational age (weeks)	20.7 (18.0–26.0)
Quintero stage	
Stage I	79 (21.8)
Stage II	93 (25.6)
Stage III	161 (44.4)
Stage IV	30 (8.3)
sFGR	
Donor	241 (66.4)
Recipient	29 (8.0)
EFW (g)	
Donor	290 (115–953)
Recipient	385 (181–1076)
Duration of laser (min)	17 (1–145)
Arterioarterial anastomoses	
Yes	70 (19.3)
No	293 (80.7)

Data are given as median (range) or *n* (%). EFW, estimated fetal weight; sFGR, selective fetal growth restriction.

For Analysis 1, the median preoperative UVF/kg of the recipient and donor was 106.0 (range, 28.0–497.5)mL/min/kg and 79.9 (range, 9.7–343.4) mL/min/kg, respectively, with a median difference between the recipient and donor of 20.12 (range, −229.5 to 424.7) mL/min/kg, *P* < 0.0001. The median preoperative UVF/kg centile of the recipient was higher than that of the donor (31.6% (range, 0–100%) *vs* 8.2% (range, 0–100%)) (Figure 3), with a median difference of 3.6% (range, −99.6% to 99.9%), *P* < 0.0001.

**Figure 3:**
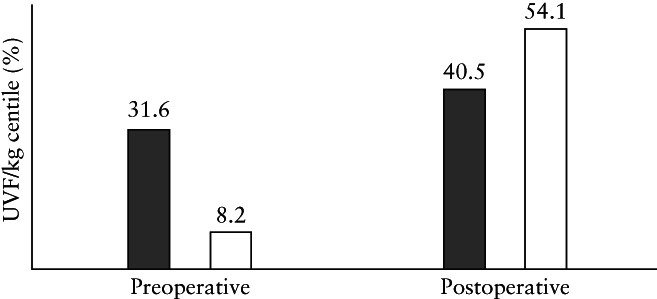
Umbilical vein flow per kg centile (UVF/kg centile) of recipient (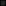) and donor (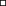) twins, preoperatively and postoperatively, in monochorionic diamniotic twin pregnancies undergoing laser surgery for twin–twin transfusion syndrome.

Bivariate analysis demonstrated an association of the preoperative differences in UVF/kg centiles with Quintero stage (Table 2) and presence of AA anastomoses (Table 3). Linear regression models with Quintero stage (Stage I used as reference) and presence of AA (absence of AA used as reference), as the independent variables, predicted the preoperative differences in UVF/kg centiles (adjusted *R* ^2^, 0.1424, *P* < 0.0001) (data not shown). When adjusted for Quintero stage and presence of AA anastomoses, the preoperative median difference in UVF/kg centiles between the recipient and donor was 17.9% (range, −17.1% to 57.6%), *P* < 0.0001.

**Table 2:** Bivariate associations of Quintero stage with difference in umbilical vein flow per kg centile (ΔUVF/kg centile) in recipient *vs* donor twin (preoperatively and postoperatively) and postoperative *vs* preoperative period (among donors and recipients)

Analysis	ΔUVF/kg centile (%)				*P*
	Stage I (*n* = 79)	Stage II (*n* = 93)	Stage III (*n* = 161)	Stage IV (*n* = 30)	
Recipient *vs* donor					
Preoperative	0.5 (−99.6 to 99.9)	1.1 (−99.1 to 98.2)	20.9 (−98.2 to 99.9)	−0.3 (−98.3 to 99.9)	< 0.0001
Postoperative	−14.3 (−98.7 to 96.6)	−5.2 (−94.6 to 99.6)	0.0 (−99.6 to 100)	−20.1 (−100 to 95.6)	0.0009
Postoperative *vs* preoperative					
Recipient	0.5 (−87.1 to 76.6)	10.5 (−99.9 to 99.5)	0.0 (−95.5 to 94.6)	0.0 (−89.2 to 72.5)	0.0015
Donor	1.3 (−86.3 to 97.0)	29.8 (−78.7 to 99.7)	14.4 (81.0 to 100)	13.3 (−82.1 to 100)	0.0124

Data are given as median (range).

**Table 3:** Bivariate associations of presence of arterioarterial vascular anastomoses (AA) with difference in umbilical vein flow per kg centile (ΔUVF/kg centile) in recipient *vs* donor twin (preoperatively and postoperatively) and postoperative *vs* preoperative period (among donors and recipients)

Analysis	ΔUVF/kg centile (%)		*P*
	AA (*n* = 70)	No AA (*n* = 293)	
Recipient *vs* donor			
Preoperative	51.5 (−98.3 to 99.9)	0.2 (−99.6 to 99.5)	< 0.0001
Postoperative	8.3 (−99.1 to 100)	−10.5 (−100 to 99.6)	< 0.0001
Postoperative *vs* preoperative			
Recipient	−4.8 (−95.5 to 76.7)	2.4 (−99.0 to 99.5)	0.0016
Donor	1.1 (−78.9 to 100)	20.6 (−86.3 to 100)	0.0400

Data are given as median (range).

The median postoperative UVF/kg of the recipient and donor was 109.8 (range, 31.6–346.5) and 110.1 (range, 24.9–741.5) mL/min/kg, respectively, with a median difference between the recipient and donor of −5.26 (range, −640.0 to 254.0) mL/min/kg, *P* = 0.1334. The median postoperative UVF/kg centile of the recipient and donor twins was 40.5% (range, 0–100%) and 54.1% (range, 0–100%), respectively, with a median difference between the recipient and donor of −5.2% (range, −100% to 100%), *P* = 0.0021. When adjusted for Quintero stage and presence of AA anastomoses, the postoperative median difference in UVF/kg centiles between the recipient and donor was −7.2% (range, −33.6% to 25.7%), *P* < 0.0001.

For Analysis 2, the change in the median UVF/kg between the postoperative and preoperative period for the recipient and donor was 5.1 (range, −343.5 to 238.1), *P* = 0.0848 and 31.6 (range, −128.3 to 562.1) mL/min/kg, *P* < 0.0001, respectively. The median UVF/kg centile of the recipient twin increased from 31.6% (range, 0–100%) to 40.5% (range, 0–100%) (Figure 3), with a median difference in UVF/kg centiles between the postoperative and preoperative period of 1.1% (range, –99.9% to 99.5%), *P* = 0.0672. The median UVF/kg centile of the donor twin increased from 8.2% (range, 0–100%) to 54.1% (range, 0–100%) (Figure 3), with a median difference of 14.1% (range, −86.3% to 100%), *P* < 0.0001.

The median difference in UVF/kg centiles between the postoperative and preoperative period for both recipients and donors was associated with Quintero stage (Table 2) and presence of AA anastomoses (Table 3) on bivariate and linear regression analyses. On linear regression analysis, the adjusted *R* ^2^ value was 0.0290 (*P* = 0.0057) for recipients and 0.0230 (*P* = 0.0150) for donors (data not shown). When adjusted for Quintero stage and presence of AA anastomoses, the median difference in recipient UVF/kg centile between the postoperative and preoperative period was 2.2% (range, −10.8% to 13.8%), *P* < 0.0001. When adjusted for Quintero stage and presence of AA anastomoses, the median difference in donor UVF/kg centile between the postoperative and preoperative period was 27.3% (range, 8.2%–34.6%), *P* < 0.0001.

## DISCUSSION

The pathophysiology of TTTS is attributed to imbalance in blood flow between monochorionic twins, which occurs due to shunting of blood through vascular communications in the common placenta. The UV is the only conduit of blood from the placenta to the fetus; therefore, any fetoplacental hemodynamic changes are reflected by its flow parameters. In TTTS, the discrepancy in UVF between the recipient and donor twins reflects the unidirectional net flow of blood from one twin to the other. The findings of this study are consistent with this theory, as recipient twins had a significantly higher UVF/kg centile compared with donors prior to laser surgery for TTTS. At the assessment 24 h after corrective laser surgery, the UVF/kg centile of recipient twins remained relatively stable, while the UVF/kg centile of donor twins increased significantly. These findings suggest rapid correction of blood‐flow imbalance following laser surgery.

Gratacós *et al*.^13^ (*n* = 32) and Ishii *et al*.^14^ (*n* = 41) directly measured time‐averaged mean velocity, also referred to as intensity‐weighted mean velocity^20^, and calculated UVF (mL/min) using the following equation: time‐averaged mean velocity (cm/s) × mean cross‐sectional area (cm^2^) × 60. In contrast, we measured TAMX to derive the time‐averaged mean velocity (TAMX × 0.5), assuming parabolic flow, and calculated UVF (mL/min) as follows: TAMX (cm/s) × 0.5 × mean cross‐sectional area (cm^2^) × 60. Figueras *et al*.^20^ describe both methods of calculating velocity and conclude that, while both have their limitations, the latter is less software‐dependent and more clearly defined.

Although our UVF/kg values are not directly comparable to those reported by Gratacós *et al*.^13^ and Ishii *et al*.^14^ because of the different methods used for calculating velocity, the patterns of change in UVF in the previous studies were similar to those observed in this study. Both previous studies reported higher median UVF/kg in recipient twins compared with donor twins before surgery (260 *vs* 151 mL/min/kg, *P* < 0.0001^13^ and 276.5 *vs* 173.2 mL/min/kg, *P* < 0.0001^14^). Gratacós *et al*.^13^ also reported that the median UVF/kg in donors increased by approximately 50% (151–232 mL/min/kg, *P* < 0.0001) 1 day after surgery, while recipients had a non‐significant postoperative increase in median UVF/kg (260–283 mL/min/kg). Likewise, Ishii *et al*.^14^ reported a significant postoperative increase in median UVF/kg of donors from 173.2 to 274.0 mL/min/kg (*P* < 0.0001) and a non‐significant change in recipients (276.5 to 282.8 mL/min/kg, *P* = 0.063), which is concordant with our findings.

Placental territory discordance is thought to be the cause of sFGR in monochorionic pregnancy^21^. In this study, 66.4% of TTTS cases were found to have sFGR in the donor twin, which is consistent with the reported previously 2/3 ratio^22^. While having an unequal share of the placenta may have an impact on UVF in theory, preoperative flow differences between the recipient and donor in this study population did not vary according to presence of donor sFGR.

The preoperative flow difference between the recipient and donor twins varied according to Quintero stage and presence of AA anastomoses. Patients with Stage‐II or ‐III TTTS had an increased preoperative difference between recipients and donors when compared to the Stage‐I TTTS group, while Stage‐IV TTTS did not have an effect. Patients with, compared to those without, AA anastomoses had a higher preoperative median difference in UVF/kg centile between the recipient and donor twins (51.5% *vs* 0.2%, *P* < 0.0001) (Table 3). In other words, the presence of AA anastomoses was associated with an increased median preoperative difference between the recipient and donor. This finding is not supportive of the hypothesis that the bidirectional nature of flow in AA anastomoses and their wide radii compensate for unequal blood flow between twins and decrease the severity of TTTS^1^, ^23^.

### Strengths and limitations

This study has several strengths. The parameter we used to compare differences in UVF (UVF/kg centile) was derived from a *Z*‐score calculator, which converted *Z*‐scores to their corresponding centiles and accounted for both GA and EFW^18^. It allowed for comparisons of centiles, which are more easily interpretable than comparisons of UVF in mL/min. Furthermore, our sample size was large, and we were able to adjust for Quintero stage and presence of AA anastomoses, both of which were associated with UVF changes. The findings of this study should be generalizable because of the large sample size and the representativeness of the study population, which came from a TTTS referral center in which procedures were performed by an experienced surgeon.

The limitations of this study include potential variability in data accuracy given the operator‐dependent nature of sonographic measurements. Different sonographers took measurements of UV parameters over a period of 10 years, and while the measurement technique was the same, operator‐dependent variability is inevitable. An additional limitation of this study is that all ultrasound UV parameters were acquired from the free‐loop portion of the UV. While this technique is in line with that utilized by Ishii *et al*.^14^ and DeVore and Epstein^18^, it is in contrast to that in other studies in which the intra‐abdominal portion of the UV was measured for the purposes of UVF analysis^13^, ^15^, ^16^, ^17^. Although both UV measurement techniques have demonstrated high intra‐ and inter‐rater reliability in a recent study^24^, free‐loop UV measurements exhibited greater variance with increasing UVF values, suggesting that the intra‐abdominal portion of the UV may be a more clinically appropriate site for UVF assessment. The *Z*‐score calculator presented a potential limitation because it was derived using a singleton‐fetus population and was applied to our twin‐fetus population. Despite the abovementioned limitations, our findings are not only concordant with prior published studies, but are also physiologically plausible.

### Conclusions

The preoperative difference in UVF between the recipient and donor twins confirmed that unbalanced intertwin blood exchange is a characteristic of TTTS. Postoperative measures that demonstrated an increased overall median UVF in donors and overall unchanged median UVF in recipients confirmed that the ablation of vascular communications resulted in rapid improvement of perfusion in donor twins and a relatively stable perfusion of recipient twins. Future studies exploring the effect of AA anastomoses and sequential surgical technique on UVF parameters may offer a better understanding of pre‐ and postoperative hemodynamic fluctuations in TTTS. The performance of the preoperative difference in UVF/kg between the recipient and donor twins in predicting TTTS, as a prediagnostic step or estimate of severity, should be investigated further to assess its potential clinical utility.

## Data Availability

The data that support the findings of this study are available from the corresponding author upon reasonable request.

## References

[1] Society for Maternal–Fetal Medicine , Simpson LL . Twin–twin transfusion syndrome. Am J Obstet Gynecol 2013; 208: 3–18. 23200164 10.1016/j.ajog.2012.10.880

[2] Quintero RA , Morales WJ , Allen MH , Bornick PW , Johnson PK , Kruger M . Staging of twin–twin transfusion syndrome. J Perinatol 1999; 19: 550–555. 10645517 10.1038/sj.jp.7200292

[3] Senat MV , Deprest J , Boulvain M , Paupe A , Winer W , Ville Y . Endoscopic laser surgery versus serial amnioreduction for severe twin‐to‐twin transfusion syndrome. N Engl J Med 2004; 351: 136–144. 15238624 10.1056/NEJMoa032597

[4] van Klink JMM , Koopman HM , van Zwet EW , Oepkes D , Walther FJ , Lapriore E . Cerebral injury and neurodevelopmental impairment after amnioreduction versus laser surgery in twin‐twin transfusion syndrome: A systematic review and meta‐analysis. Fetal Diagn Ther 2013; 33: 81–89. 22922370 10.1159/000341814

[5] Roberts D , Neilson JP , Kilby MD , Gates S . Interventions for the treatment of twin–twin transfusion syndrome. Cochrane Database Syst Rev 2014; 1: CD002073. 10.1002/14651858.CD002073.pub3 PMC10816955 24482008

[6] Di Mascio D , Khalil A , D'Amico A , Buca D , Benedetti Panici P , Flacco ME , Manzoli L , Liberati M , Nappi L , Berghella V , D'Antonio F . Outcome of twin–twin transfusion syndrome according to Quintero stage of disease: systematic review and meta‐analysis. Ultrasound Obstet Gynecol 2020; 56: 811–820. 32330342 10.1002/uog.22054

[7] Khalil A , Cooper E , Townsend R , Thilaganathan B . Evolution of stage 1 twin‐to‐twin transfusion syndrome (TTTS): systematic review and meta‐analysis. Twin Res Hum Genet 2016; 19: 207–216. 27137946 10.1017/thg.2016.33

[8] Emery SP , Hasley SK , Catov JM , Miller RS , Moon‐Grady AJ , Baschat AA , Johnson A , Lim FY , Gagnon AL , O'Shaughnessy RW , Ozcan T , Luks FI . North American Fetal Therapy Network . North American Fetal Therapy Network: intervention *vs* expectant management for stage I twin‐twin transfusion syndrome. Am J Obstet Gynecol 2016; 215: 346.e1–7. 10.1016/j.ajog.2016.04.024 27131587

[9] Stirnemann J , Slaghekke F , Khalek N , Winer N , Johnson A , Lewi L , Massoud M , Bussieres L , Aegerter P , Hecher K , Senat MV , Ville Y . Intrauterine fetoscopic laser surgery versus expectant management in stage 1 twin‐to‐twin transfusion syndrome: an international randomized trial. Am J Obstet Gynecol 2021; 224: 528.e1–12. 10.1016/j.ajog.2020.11.031 33248135

[10] Rüegg L , Hüsler M , Krähenmann F , Natalucci G , Zimmermann R , Ochsenbein‐Kölble N . Outcome after fetoscopic laser coagulation in twin–twin transfusion syndrome – is the survival rate of at least one child at 6 months of age dependent on preoperative cervical length and preterm prelabour rupture of fetal membranes? J Matern Fetal Neonatal Med 2020; 33: 852–860. 30196741 10.1080/14767058.2018.1506441

[11] Chmait RH , Kontopoulos EV , Korst LM , Llanes A , Petisco I , Quintero RA . Stage‐based outcomes of 682 consecutive cases of twin‐twin transfusion syndrome treated with laser surgery: the US Fetus experience. Am J Obstet Gynecol 2011; 204: 393.e1–6. 10.1016/j.ajog.2011.02.001 21411051

[12] Assaf SA , Korst LM , Chmait RH . Normalization of amniotic fluid levels after fetoscopic laser surgery for twin‐twin transfusion syndrome. J Ultrasound Med 2010; 29: 1431–1436. 20876896 10.7863/jum.2010.29.10.1431

[13] Gratacós E , Van Schoubroeck D , Carreras E , Devlieger R , Roma E , Cabero L , Deprest J . Impact of laser coagulation in severe twin–twin transfusion syndrome on fetal Doppler indices and venous blood flow volume. Ultrasound Obstet Gynecol 2002; 20: 125–130. 12153662 10.1046/j.1469-0705.2002.00749.x

[14] Ishii K , Chmait RH , Martínez JM , Nakata M , Quintero RA . Ultrasound assessment of venous blood flow before and after laser therapy: approach to understanding the pathophysiology of twin–twin transfusion syndrome. Ultrasound Obstet Gynecol 2004; 24: 164–168. 15287054 10.1002/uog.1104

[15] Yamamoto M , Nasr B , Ortqvist L , Bernard J‐P , Takahashi Y , Ville Y . Intertwin discordance in umbilical venous volume flow: a reflection of blood volume imbalance in twin‐to‐twin transfusion syndrome. Ultrasound Obstet Gynecol 2007; 29: 317–320. 17323309 10.1002/uog.3959

[16] Gungor S , Glosemeyer P , Huber A , Hecher K , Baschat AA . Umbilical venous volume flow in twin–twin transfusion syndrome. Ultrasound Obstet Gynecol 2008; 32: 800–806. 18837441 10.1002/uog.6227

[17] Baschat AA , Gungor S , Glosemeyer P , Huber A , Hecher K . Changes in umbilical venous volume flow after fetoscopic laser occlusion of placental vascular anastomoses in twin‐to‐twin transfusion syndrome. Am J Obstet Gynecol 2010; 203: 479.e1–6. 10.1016/j.ajog.2009.11.013 20864074

[18] DeVore G , Epstein A . Computing Z‐score equations for clinical use to measure fetal umbilical vein size and flow using six independent variables of age and size. J Ultrasound Med 2021; 41: 1949–1960. 34792203 10.1002/jum.15872

[19] Gomez N , Monson M , Chon A , Korst L , Llanes A , Chmait R . Outcomes of laser surgery for stage I twin–twin transfusion syndrome. Prenat Diagn 2022; 42: 172–179. 35032038 10.1002/pd.6094

[20] Figueras F , Fernández S , Hernández‐Andrade E , Gratacós E . Umbilical venous blood flow measurement: accuracy and reproducibility. Ultrasound Obstet Gynecol 2008; 32: 587–591. 18618412 10.1002/uog.5306

[21] Wang X , Li L , Yuan P , Zhao Y , Wei Y . Comparison of placental characteristics of twin–twin transfusion syndrome with and without selective intrauterine growth restriction. J Matern Fetal Neonatal Med 2020: 1–7. 33203261 10.1080/14767058.2020.1849110

[22] Van Winden K , Quintero R , Kontopoulos E , Korst L , Llanes A , Chmait R . Perinatal survival in cases of twin–twin transfusion syndrome complicated by selective intrauterine growth restriction. J Matern Fetal Neonatal Med 2015; 28: 1549–1553. 25184748 10.3109/14767058.2014.960834

[23] Tan T , Taylor M , Wee L , Vanderheyden T , Wimalasundera R , Fisk N . Doppler for artery–artery anastomosis and stage‐independent survival in twin‐twin transfusion. Obstet Gynecol 2004; 103: 1174–1180. 15172849 10.1097/01.AOG.0000127881.34144.d8

[24] Kanazawa S , Muromoto J , Ozawa K , Mikami M , Ogawa K , Wada S , Sago H . Reliability and characteristics of ultrasound measurement of fetal umbilical venous blood flow volume according to the site of measurement. J Med Ultrason *(* 2001) 2020; 47: 305–312. 10.1007/s10396-019-00999-3 31912321

